# Branding and a child’s brain: an fMRI study of neural responses to logos

**DOI:** 10.1093/scan/nss109

**Published:** 2012-12-14

**Authors:** Amanda S. Bruce, Jared M. Bruce, William R. Black, Rebecca J. Lepping, Janice M. Henry, Joseph Bradley C. Cherry, Laura E. Martin, Vlad B. Papa, Ann M. Davis, William M. Brooks, Cary R. Savage

**Affiliations:** ^1^Department of Psychology, University of Missouri-Kansas City, 5030 Cherry Street, Kansas City, MO 64110, MO, USA, ^2^Center for Children’s Healthy Lifestyles and Nutrition, University of Kansas Medical Center/Children’s Mercy Hospital, 610 E. 22nd Street, Kansas City, MO 64108, KS, USA, ^3^Center for Health Behavior Neuroscience, University of Kansas Medical Center, 3901 Rainbow Boulevard, Kansas City, KS 66160, KS, USA, ^4^Department of Psychology, University of Kansas, 1415 Jayhawk Blvd., Fraser Hall, Room 426, Lawrence, KS 66045-7556, KS, USA, ^5^Hoglund Brain Imaging Center, University of Kansas Medical Center, 3901 Rainbow Boulevard, Kansas City, KS 66160, KS, USA, ^6^Department of Preventive Medicine, University of Kansas Medical Center, 3901 Rainbow Boulevard, Kansas City, KS 66160, KS, USA, ^7^Department of Pediatrics, University of Kansas Medical Center, 3901 Rainbow Boulevard, Kansas City, KS 66160, KS, USA, ^8^Department of Neurology, University of Kansas Medical Center, 3901 Rainbow Boulevard, Kansas City, KS 66160, KS, USA and ^9^Department of Psychiatry, University of Kansas Medical Center, 3901 Rainbow Boulevard, Kansas City, KS 66160, KS, USA

**Keywords:** children, brands, fMRI, prefrontal cortex, neuromarketing, food logos

## Abstract

Branding and advertising have a powerful effect on both familiarity and preference for products, yet no neuroimaging studies have examined neural response to logos in children. Food advertising is particularly pervasive and effective in manipulating choices in children. The purpose of this study was to examine how healthy children’s brains respond to common food and other logos. A pilot validation study was first conducted with 32 children to select the most culturally familiar logos, and to match food and non-food logos on valence and intensity. A new sample of 17 healthy weight children were then scanned using functional magnetic resonance imaging. Food logos compared to baseline were associated with increased activation in orbitofrontal cortex and inferior prefrontal cortex. Compared to non-food logos, food logos elicited increased activation in posterior cingulate cortex. Results confirmed that food logos activate some brain regions in children known to be associated with motivation. This marks the first study in children to examine brain responses to culturally familiar logos. Considering the pervasiveness of advertising, research should further investigate how children respond at the neural level to marketing.

## INTRODUCTION

Advertising is a dominant industry in the United States with food and beverage companies alone spending more than $10 billion annually to market their products to children (Institute of Medicine Committee, 2006). The intense marketing toward youth is driven by companies’ ambitions for brand recognition, preference and loyalty. The average child in the United States views more than 5500 television food advertisements per year (Federal Trade Commission, 2007). Of these, 98% are for products high in fat, sugar and/or sodium (Powell *et al.*, 2007). Advertising is successful with studies on the effects of television food advertising showing that children exposed to advertisements will prefer advertised foods at much higher rates than children who were not exposed (Coon and Tucker, 2002). Also, the amount of exposure children have to food advertisements directly impacts the number of attempts they make to influence their parents’ purchases (Coon and Tucker, 2002). Robinson et al. (2007) asked children aged 3–5 years to taste identical foods and beverages labeled in McDonald’s™ or unbranded packaging. Although the food and drink samples were identical, children indicated a statistically significant preference for the taste of food and drinks labeled with McDonald’s™ brand logos, exemplifying how food advertising impacts children’s preferences and food motivation. The consensus among published reviews is that ‘food promotion has a causal and direct effect on children’s food preferences, knowledge, and behavior’ (Livingstone, 2005: 283). In addition, some experts have cited food marketing as one of the contributors to the recent rise in childhood obesity (Harris *et al.*, 2009).

Neuroimaging techniques such as functional magnetic resonance imaging (fMRI) can help to improve understanding of how people process, evaluate and respond to product brands (see Plassman *et al.*, 2012 for a review). Published neuromarketing studies of healthy adults viewing culturally familiar logos have determined that the prefrontal cortex (PFC) and hippocampus are involved in brand recognition. Specifically, product brands activate dorsolateral PFC, ventromedial PFC, orbitofrontal cortex (OFC), anterior cingulate cortex (ACC), ventral striatum and hippocampus (e.g. McClure *et al.*, 2004; Schaefer *et al.*, 2006; Schaefer and Rotte, 2007a,b; Schaefer and Rotte, 2011; Esch *et al.*, 2012). Moreover, the PFC, OFC, ACC, ventral striatum and hippocampus have also been identified as being involved in food motivation, reward processing and general appetitive cues (as both ‘drive’ and ‘control’ regions) (e.g. Gautier *et al.*, 2000; Small *et al.*, 2001; DelParigi *et al.*, 2005; Simmons *et al.*, 2005; Martin *et al.*, 2010).

Studies on children’s brain responses to actual food images have implicated similar brain regions as those identified in adults (Holsen *et al.*, 2005; Killgore & Yurgelun-Todd, 2005; Bruce *et al.*, 2010; Davids *et al.*, 2010). In healthy weight children, one fMRI study compared brain activations in response to appetizing food images when children were hungry and when they were satiated (Holsen *et al.*, 2005). Increased activations to food images were reported in insula, amygdala, medial frontal cortex and OFC, which are similar to adult findings. Another study compared adolescent and adult brain activation and identified increased activation to food images in OFC and hippocampus (Killgore & Yurgelun-Todd, 2005). The neural networks associated with food motivation are the same regions discussed in the well-supported theory of brain development (Casey *et al.*, 2000). This theoretical model posits that the increase in risk-taking behavior in adolescence is attributed to uneven neurobiological development in brain regions associated with cognitive control and emotional drive (Somerville and Casey, 2010). Specifically, reward regions including striatum mature before the cognitive and self-control regions of the PFC. Therefore, without the necessary inhibitory processes to aid in decision making, youth are particularly susceptible to making poor health behavior choices and these differences may be particularly pronounced when evaluating appetitive cues (Somerville and Casey, 2010).

Despite recent interest in neuromarketing and the neuroscience of food motivation, no studies thus far have examined brain activation in children viewing brand logos. Therefore, the aim of this study was to examine neural responses to product brands in children to gain a better understanding of how children’s brains respond to appetitive cues frequently used in advertising. We hypothesized an increase in activity in the limbic and paralimbic system, including ventral striatum, and prefrontal brain regions when children were viewing food logos compared with either non-food logos or a baseline condition. We used an fMRI stimulus paradigm including familiar food and non-food logos that were common in the United States, e.g. McDonald’s arches®, Lucky Charms™ leprechaun, Rice Krispies™ elves *vs* the Target™ bulls-eye, the Energizer Bunny®, FedEx® *vs* blurred images of logos matched on color composition and brightness (baseline condition) as comparisons. A better understanding of children’s responses to food logos will be beneficial in elucidating the complex relationships between advertising and neural responses to motivational cues.

## MATERIALS AND METHODS

The protocols for the pilot validation study and the main fMRI study were approved by the Human Subjects Committee at the University of Kansas Medical Center (KUMC). Written informed consent was obtained from each child’s parent/legal guardian and written informed assent was obtained from each child before study participation.

### Validation of logo stimuli

**Fig. 1:**
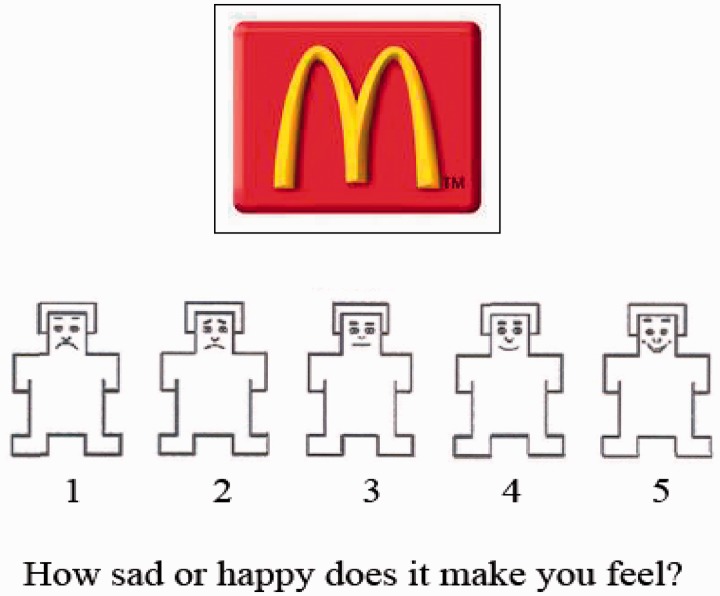
Example of item from the pilot validation of logo stimuli prior to the main fMRI study.

A validation study was first conducted to select the most appropriate logos for use in the activation paradigm. Thirty-two participants (13 males) aged 9–16 years (mean = 11.5 years, s.d. = 2.2) rated 239 culturally familiar brand logos on a five-point Likert scale on three categories: familiarity, valence (happy/sad) and arousal (exciting/boring) (Figure 1). This standardized scale has been used in many stimulus validation studies for the International Affective Picture Set (Lang *et al.*, 2008). Based on the participants’ ratings, 60 food and 60 non-food logos that were high on familiarity were selected (see online Supplementary Data). Food logos as a group were matched on familiarity with non-food logos [*t*(118) = 0.33, *P* = 0.74]. The food and non-food logos were not significantly different on valence [*t*(118) = 1.26, *P*= 0.21] or arousal [*t*(118) = 1.49, *P* = 0.14]. These 120 logos were used in the fMRI paradigm in the main study described later. Baseline images were created from the food and non-food logos using three iterations of a fast Fourier transform to render the logos unidentifiable. The baseline images were therefore matched to the food and non-food logos on visual properties of color composition and brightness.

### Main fMRI study

#### Participants

Seventeen children (10 males) with a mean age of 11.8 years (s.d. = 1.4, range 10–14) were recruited from broadcast email messages sent to the KUMC employees and from the pediatric clinic. All participants were in age-appropriate grades. Exclusion criteria included major psychiatric diagnoses and neurological illness (parental interview), left-handedness and impaired, uncorrected vision. All participants spoke English as their primary language. None of these participants took part in the validation study.

#### Procedures and methods

After informed consent was obtained, participants and their parents completed demographic measures. Time since last food intake was at least 4 h. Prior to the scan, the MRI experience was fully explained to the children and their parents. The scanning session took ∼45 min.

#### fMRI data acquisition

Data were acquired with a 3-Tesla Siemens Allegra scanner. Each scan consisted of one anatomical and two 6 min 36 s functional sequences. T_1_-weighted 3D MPRAGE anatomic images were acquired [time to repetition (TR)/time to echo (TE) = 23/4 ms, flip angle = 8°, field of view = 256 mm, matrix = 256 × 192 and slice thickness = 1 mm]. Gradient-echo blood-oxygen-level-dependent scans were acquired in 43 contiguous axial slices at a 40° angle to the anterior commissure-posterior commissure (AC–PC) line [TR/TE = 3000/30 ms, slice thickness = 3 mm (0.5 mm skip), in-plane resolution = 3 × 3 mm, 130 data points]. To optimize signal in ventromedial prefrontal regions, the susceptibility artifact was addressed in two ways: (i) acquiring the slices at a 40° angle to the AC–PC line and (ii) positioning all participants in the scanner so that the angle of the AC–PC plane was between 17° and 22° from the axial plane in scanner coordinate space. This procedure also standardized head positioning.

#### Experimental paradigm

A block design with two functional runs (each run was 6 min 36 s) was used to display the food logos, non-food logos and blurred baseline images (Bruce *et al.*, 2010). Each logo was presented only once to each participant. Functional scans involved three repetitions of each block of stimulus type (i.e. each block contained 10 food logos or 10 non-food logos), alternated between blocks of 10 blurred images. Stimulus presentation time was 2.5 s with an interstimulus interval of 0.5 s. The order of category presentation was counterbalanced across participants. Visual images were back-projected to a screen mounted on the back of the MRI scanner, and participants viewed the images through a mirror on the head coil. Foam cushions were placed around the participants’ heads to minimize movement.

#### fMRI data analysis

Data preprocessing and statistical analyses were conducted using BrainVoyager QX 2.1 statistical package (Brain Innovation, Maastricht, the Netherlands). The preprocessing steps included trilinear 3D motion correction, sinc-interpolated slice scan time correction, 2D spatial smoothing (4 mm Gaussian filter) and high-pass filter temporal smoothing. Functional images were realigned to the anatomic images obtained within each session and normalized to the BrainVoyager template image, which conforms to the space defined by the Talairach and Tournoux stereotaxic atlas (Talairach and Tournoux, 1988). Four runs out of 34 (two runs each from 17 participants) were discarded due to motion >3 mm of movement on an axis (*x*, *y* and *z*).

Activation maps were generated using statistical parametric methods and random effects in BrainVoyager QX. Statistical contrasts were conducted using multiple regression analysis with the general linear model allowing multiple predictors to be built into the model. Regressors representing experimental conditions of interest were modeled with a hemodynamic response filter and entered into the multiple regression analysis using a random-effects model. Contrasts between conditions of interest were assessed with *t*-statistics across whole brain. For each contrast (food logo *vs* baseline, non-food logo *vs* baseline and food logo *vs* non-food logo), voxel values were considered significant if the activation survived a statistical cluster-based threshold of *P* < 0.01, corrected. We corrected for multiple comparisons using the familywise approach (α < 0.05; *P* < 0.01, *k* = 9 voxels), determined by Monte Carlo simulation in BrainVoyager (Goebel *et al.*, 2006; Lieberman and Cunningham, 2009).

## RESULTS

### Food logos *vs* baseline

**Fig. 2:**
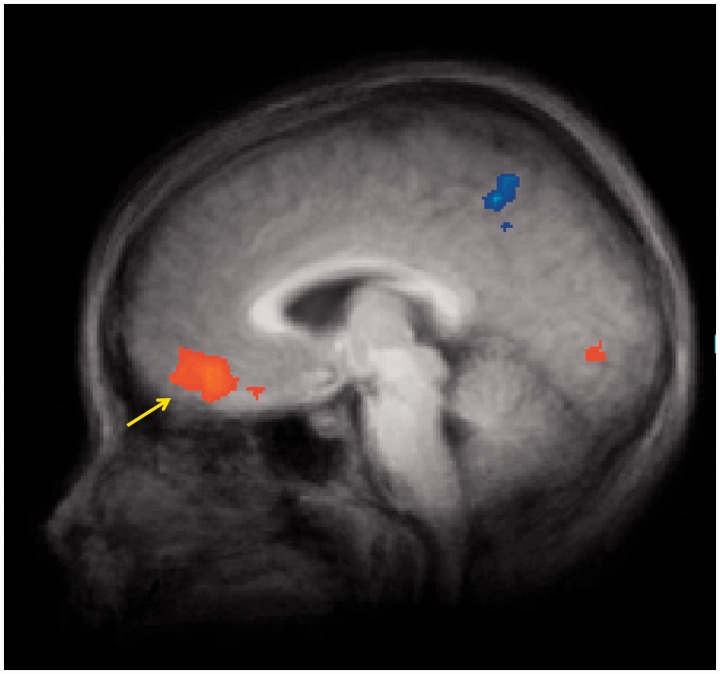
fMRI statistical maps (sagittal perspective) showing results from food logo *vs* baseline contrasts, co-registered with average structural MRI data from participants. Significance thresholds are set at *P *< 0.01, corrected (cluster threshold = 9 voxels). Arrow highlights greater activation in OFC.

As shown in Table 1, the food logos *vs* baseline analysis (*P* < 0.01, corrected) revealed significant activations in left OFC [Brodmann’s area (BA) 10/11] (Figure 2) and bilateral inferior frontal gyrus (IFG, BA 13), left temporal cortex and bilateral visual cortex. Significant deactivations to food logos were found in right parietal, bilateral temporal and left posterior cingulate.

**Table 1:** Regions reaching significance for the contrasts between food and non-food logo stimuli in comparison to baseline images

Contrast and region	Coordinates			*t*-Value	Contiguous voxels
	*x*	*y*	*z*		
Food logos *vs* baseline					
OFC (L), BA 10/11	−6	41	−8	5.96	114
Bilateral IFG, BA 13	−39	29	10	6.78	280
BA 47	24	29	−2	6.19	15
Bilateral occipital cortex, BA 18	27	−82	1	8.43	1326
	−27	−79	−11	11.47	1547
Bilateral temporal cortex	−51	−34	1	6.36	11
	−57	−16	−17	5.52	12
	−48	−22	13	−5.01	30
	39	−10	−5	−3.92	12
Parietal cortex (R), BA 40	63	−37	31	−5.44	90
Posterior cingulate (L), BA 31	−6	−46	43	−6.02	183
Non-food logos *vs* baseline					
Medial prefrontal (L), BA 6	−9	5	55	6.82	10
IFG (L), BA 13	−42	26	10	5.71	119
Thalamus (R)	21	−25	4	4.82	14
Bilateral fusiform gyrus, BA 19/37	39	−70	−11	9.88	1328
	−39	−46	−17	11.28	1445
Superior frontal gyrus (R), BA 10/9	30	68	1	−4.21	11
	30	59	28	−4.63	11
Insula (L), BA 13	−42	−10	4	−5.86	63
Insula/temporal cortex (L)	−45	−31	19	−7.01	19
Precuneus (R), BA 30/31	15	−55	16	−4.60	13
	6	−37	43	−5.75	372
Temporal cortex (R), BA 21	54	−22	−2	−5.44	20
Bilateral parietal cortex	63	−28	43	−5.38	180
	−63	−28	37	−5.05	87

*P *< 0.01, cluster corrected at 9 voxels. Activations are listed first (positive *t*-values) followed by deactivations (negative *t*-values) for each contrast. L = left; R = right.

### Non-food logos *vs* baseline

The non-food logos *vs* baseline analysis (*P* < 0.01, corrected) revealed significant activations in left medial PFC, left IFG, right thalamus and bilateral fusiform gyrus (Table 1). Significant deactivations to non-food logos were found in right superior frontal gyrus, left insula/temporal cortex, bilateral parietal cortex, right temporal cortex and right precuneus.

### Food logos *vs* non-food logos

**Fig. 3:**
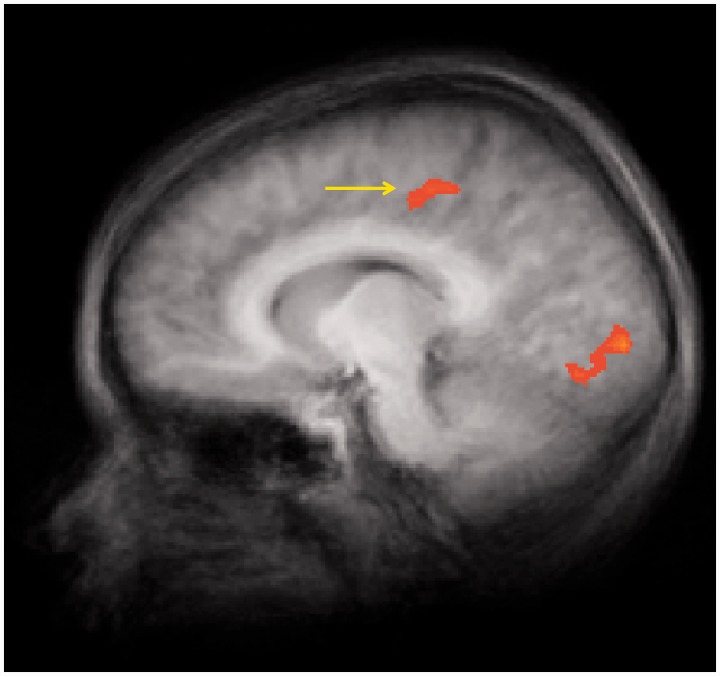
fMRI statistical maps in the sagittal view showing results from food *vs* non-food logo contrasts, co-registered with average structural MRI data from participants. Significance thresholds are set at *P *< 0.01, corrected (cluster threshold = 9 voxels). Arrow highlights greater activation in PCC.

The food logos *vs* non-food logos analysis (*P* < 0.01, corrected) revealed significant activations in right occipital cortex and right paracentral lobule and left parietal and left lingual gyrus (Table 2). Activation in right paracentral lobule extended into posterior cingulate cortex (PCC) (*P* < 0.01; *x* = 9, *y* = −23, *z* = 43) (Figure 3). No regions showed significantly greater activations to non-food compared with food logos.

**Table 2:** Regions reaching significance for the contrasts food logo stimuli in comparison to non-food logo stimuli

Contrast and region	Coordinates			*t*-Value	Contiguous voxels
	*x*	*y*	*z*		
Food logos > non-food logos					
Occipital cortex (R), BA 18	18	−85	11	6.23	71
Lingual gyrus (L), BA 17	−6	−88	−2	5.73	89
Paracentral/PCC (R), BA 31	9	−28	46	4.41	18
Parietal cortex (L), BA 40	−24	−40	55	4.85	13

*P *< 0.01, cluster corrected at 9 voxels. There were no regions where non-food logos > food logos. L = left; R = right.

## DISCUSSION

Although a growing body of neuroimaging literature documents adult brain responses to product brands, this is the first study to examine children’s brain responses to culturally familiar food and non-food logos. In healthy children, food and non-food logos activated object identification regions of the brain (visual cortex/ventral stream). Studies examining adults’ brain responses to logos also noted significant activation in these regions (Plassman *et al.*, 2012).

We found that healthy children’s brains show significant activation to food logos compared to baseline images in regions associated with both motivational value (OFC, BA 10/11) and cognitive control (IFG, BA 13). The non-food logos compared to baseline activated inferior frontal and medial PFC and thalamus. In a direct comparison between food logos and non-food logos, food logos resulted in greater activation in occipital and parietal cortex and PCC. PCC was significantly deactivated to food logos and non-food logos, only more so to non-food logos. PCC is known to be an integral member in the default mode network (Fransson and Marrelec, 2008) and it is possible the deactivations in PCC may indicate the children’s engagement with the visual stimuli. Furthermore, the food logos showed significant positive activations in occipital cortex compared with non-food logos. Other studies have shown that food images elicit brain activations in visual cortex (Simmons *et al.*, 2005). No areas were significantly more active to non-food logos *vs* food logos. Food logos may attract children’s attention more than non-food logos. This is significant considering the vast majority of foods marketed to children are for unhealthy, calorically dense foods (Powell *et al.*, 2007). Results from this preliminary study should be interpreted using the usual caution pertinent to reverse inference, and may serve as the basis for future hypothesis testing. Researchers should directly compare neural responses to food logos compared to actual images of food.

Our results in children overlap partially with findings from previous studies examining healthy adults’ brain responses to logos including significant activations in medial PFC, inferior PFC, OFC and visual cortex (Plassman *et al.*, 2012). However, unlike the adult studies, we did not observe significant activations in hippocampus or ventral striatum (caudate, nucleus accumbens). Our results are consistent with those found by previous neuroimaging studies examining children’s brain activation in response to actual food images that show activation in PFC and OFC (Holsen *et al.*, 2005; Bruce *et al.*, 2010; Davids *et al.*, 2010).

As this is the first study to examine children’s brain responses to brands, there are some limitations of the results. First, our sample size is relatively small. Future studies with larger samples would permit examination of age and gender effects in response to brands. Second, our study was limited to healthy children and the effects of advertising on obese children were not examined. Given that children are exposed to unhealthy food more often than healthy food (Klepp *et al.*, 2007), such advertising effects may have implications for childhood obesity. Research should examine the differences between healthy weight and obese children’s responses to brands. Third, because we needed to match the food and non-food logos on familiarity, valence and intensity, the logos we chose for the imaging paradigm were not the most familiar, most positively valenced food logos. Thus, findings may underemphasize the effects of food logos on children’s brain responses. Future studies wishing to further clarify the relationship between brain responses to food logos and children’s perceptions of those logos could ask participants to rate the logos while in the scanner. Finally, because we asked participants to refrain from eating for 4 h before the scan to standardize hunger, it is possible that the observed differences in brain activations between food and non-food logos could be due to hunger. Future research should consider manipulating hunger levels of participants to determine whether there is a relationship between brain responses to food logos and food motivation.

From a developmental perspective, these early findings are important, as the brain regions involved in food motivation, reward processing, decision making and self-control change throughout childhood and adolescence (Bruce *et al.*, 2011). A recently published study examined decision making in an intertemporal choice task ($20 now *vs* $50 in 10 days) using fMRI in conjunction with brand exposure. When a brand logo was subliminally presented to adults before making their choices, preferences shifted to a more immediate reward (Murawski *et al.*, 2012). The prospect of brand exposure altering decision making even in an unrelated task is compelling and worthy of further investigation. Future studies should directly compare youth of different ages and adults to determine how differential maturity affects responses to marketing and decision making regarding food and non-food products.

Children’s brains show responses to brand logos in similar regions as adults’ brains. Food logos, however, seem to be more emotionally salient than the non-food logos, perhaps due to the survival salience of food as a biological necessity. Additional research is needed to better characterize children’s brain responses to marketing and marketing’s impact on their choices and behavior.

## SUPPLEMENTARY DATA

Supplementary data are available at *SCAN* online.

## Conflict of Interest

None declared.

## Supplementary Material

Supplementary Data
