# Supplementary material for: Branding and a child’s brain: an fMRI study of neural responses to logos
Source: Soc Cogn Affect Neurosci. 2012 Dec 14;9(1):118–22. doi: 10.1093/scan/nss109 (PMC3871732; doi:10.1093/scan/nss109)
Supplement: Supplementary Data [file supp_9_1_118__index.html]

Supplementary Data 

# Branding and a child’s brain: an fMRI study of neural responses to logos

## Supplementary Data

files

**Files in this Data Supplement:**

- Supplementary Data - doc file
