# Supplementary material for: Branding and a child’s brain: an fMRI study of neural responses to logos
Source: Soc Cogn Affect Neurosci. 2012 Dec 14;9(1):118–22. doi: 10.1093/scan/nss109 (PMC3871732; doi:10.1093/scan/nss109)
Supplement: Supplementary Data [file supp_nss109_scan-12-133-File004.doc]

**Supplemental Table 1**. Food and nonfood logos in

fMRI paradigm, sorted by familiarity (highest at top)

| drpepper.bmp  pizzahut.bmp  cokebottle.bmp  wendys.bmp  cheeto.bmp  dominos.bmp  McDM.bmp  starbucks.bmp  tacobell.bmp  7up.bmp  bk.bmp  crunch.bmp  doritos.bmp  ljs.bmp  mug.bmp  oreo.bmp  sonic.bmp  fritos.bmp  ritz.bmp  trix.bmp  wonder.bmp  chipsahoy.bmp  yoplait.bmp  dunkindonuts.bmp  jellybelly.bmp  tgifridays.bmp  kfc.bmp  luckycharms.bmp  mrpeanut.bmp  nesquik.bmp  pepsi.bmp  quakerlogo.bmp  tostitos.bmp  aquafina.bmp  caprisun.bmp  chilis.bmp  lays.bmp  snapcracklepop.bmp  v8.bmp  blowpop.bmp  frostedflakes.bmp  greengiant.bmp  nestea.bmp  pillsbury.bmp  ricekrispees.bmp  keeblerelf.bmp  ruffles.bmp  minutemaid.bmp  capncrunch.bmp  dasani.bmp  dole.bmp  cheeriosbee.bmp  chefboy.bmp  wheatthins.bmp  dannon.bmp  folgers.bmp  crackerjack.bmp  hostess.bmp  kraft.bmp  triscuit.bmp | lego.bmp  honda.bmp  spongebob.bmp  FedEx.bmp  hallmark.bmp  ku.bmp  flag.bmp  winnie the pooh.bmp  garfield.bmp  mickey mouse.bmp  bestbuy.bmp  pokemon.bmp  oldnavy.bmp  sprint.bmp  wb.bmp  crayola.bmp  crest.bmp  Lowes.bmp  nike.bmp  nintendo.bmp  Playstation.bmp  windows.bmp  goodyear.bmp  redcross.bmp  verizon.bmp  abc.bmp  cathat.bmp  dell.bmp  duracell.bmp  elmo.bmp  energizer.bmp  lionking.bmp  nfl.bmp  bugsbunny.bmp  nba.bmp  royals.bmp  snoopy.bmp  VISA.bmp  elmers glue.bmp  dodge.bmp  DVD.bmp  mlb.bmp  bartsimpson.bmp  disneycastle.bmp  dove.bmp  tommyhilfiger.bmp  chevy.bmp  colgate.bmp  jeep.bmp  directTV.bmp  gap.bmp  Mizzou.bmp  kstate.bmp  kermit.bmp  nbc.bmp  walmart.bmp  bp.bmp  mercedes.bmp  bmw.bmp  puma.bmp |
| --- | --- |
